# Biotransformation of Ginsenoside Rf to Rh_1_ by Recombinant β-Glucosidase

**DOI:** 10.3390/molecules14062043

**Published:** 2009-06-08

**Authors:** Chang-Chun Ruan, Hao Zhang, Lian-Xue Zhang, Zhi Liu, Guang-Zhi Sun, Jun Lei, Yu-Xia Qin, Yi-Nan Zheng, Xiang Li, Hong-Yu Pan

**Affiliations:** 1 College of Chinese Medicinal Materials, Jilin Agricultural University, Changchun, 130118, China; E-mails: yuan.changchun1@gmail.com (C-C.R.), leijun3000@tom.com (J.L.), zhenyinan@tom.com (Y-N.Z.); 2 Institute of Agricultural Modernization, Jilin Agricultural University, Changchun, 130118, China; E-mails: gzsun1967@yahoo.com (G-Z.S.), jlndxdhs@126.com (Z.L.); 3 School of Resources and Environment, Jilin Agricultural University, Changchun, 130118, China; E-mail: haozhang100@163.com (H.Z.); 4 Engineering Research Center of Bioreactor and Pharmaceutical Development, Ministry of Education, Jilin Agricultural University, Changchun, 130118, China; E-mail: yxqin2000@126.com (Y-X.Q.); 5 Saskatoon Research Center, Agriculture and Agri-food Canada, Saskatoon, SK S7N 0X2, Canada; 6 College of Plant Science, Jilin University, Changchun, 130062, China

**Keywords:** *Aspergillus niger*, β-glucosidase, ginsenoside Rf, ginsenoside Rh_1_

## Abstract

An *Aspergillus niger* strain was isolated from the soil around ginseng fruit. *In vitro* enzyme assays showed that this strain had the ability to transform total ginsenosides (TGS) into several new products. In a further biochemical study, a β-glucosidase gene isolated from this strain, *bgl1*, was expressed in *Saccharomyces cerevisiae*. His-tagged BGL1 protein (~170 kD) showed the ability to transform ginsenoside Rf into Rh_1_.

## Introduction

**Figure 1:**
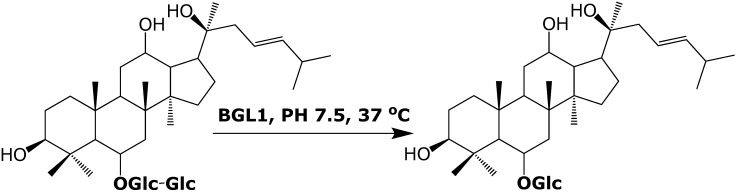
Scheme of biotransformation from ginsenoside Rf to Rh_1_ catalyzed by BGL1.

The root of *Panax ginseng* C.A. MEYER is frequently used in China as a traditional medicine [1]. Ginsenosides, as the major components of ginseng, have been reported to show various biological activities, *eg.* anti-tumor, anti-inflammatory, immune-modulatory and anti-aging effects [2,3,4,5]. Among the 30 previous reported ginsenosides, Rg_3_, compound K (CK) and Rh_1_ showed highly cytotoxicity against tumor cells [6,7,8,9]. CK was proven to be produced by intestinal microorganisms after oral administration of Rg_3_, and then further esterified to sustain it longer in the body [10,11]. During our continued work on bioactive ginsenosides, an *Aspergillus niger* strain was isolated from the soil around ginseng fruit. *In vitro* enzyme assays showed that this strain had the ability to transform total ginsenosides (TGS) into several new products [12]. In the subsequent biochemical study, a β-glucosidase gene isolated from this strain, *bgl1*, showed the ability to transform ginsenoside Rf to Rh_1_ (Figure 1).

## Results and Discussion

### Expression and purification of BGL1 in Saccharomyces cerevisiae

**Figure 2:**
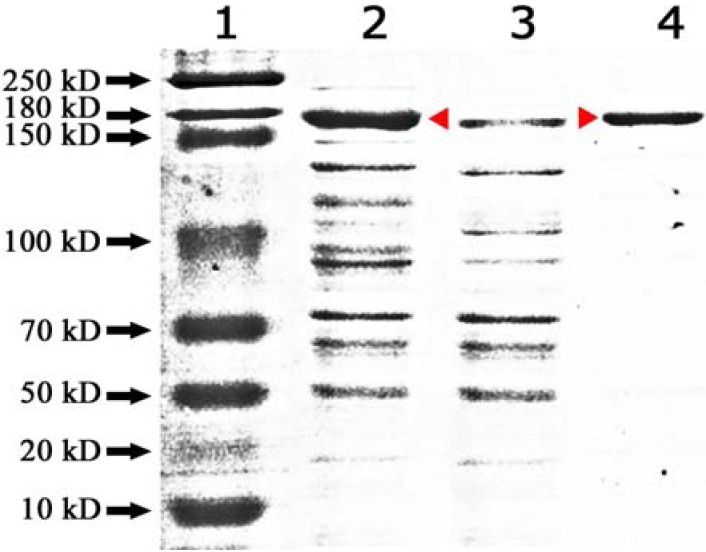
SDS-PAGE gel of expression and purification of BGL1. Lane 1: Protein marker, 2: Over-expression of BGL1; 3: Empty control; 4: Purified protein.

In the present study, the *bgl1* gene isolated from an *Aspergillus niger* strain, which encodes a glucosidase, was cloned into the yeast shuttle vector pRS423 and introduced into *Saccharomyces cerevisiae* (MGY70). SDS-PAGE analysis showed strong expression of a ~170 kDa his-tagged BGL1 protein at 37 ºC (Figure 2, lane 2). The empty pRS423 vector was used as control (Figure 2, lane 3). Soluble recombinant protein purified from cultures grown at 37ºC by his-tagging yielded a single distinct band after SDS-PAGE (Figure 2, lane 4). After dialysis, the purified recombinant BGL1 was quantified at 0.9 µg µL^-1 ^(total of 5.1 mg from 6 g bacteria cell pellet).

### In vitro biotransformation of ginsenoside Rf to Rh_1_

**Figure 3:**
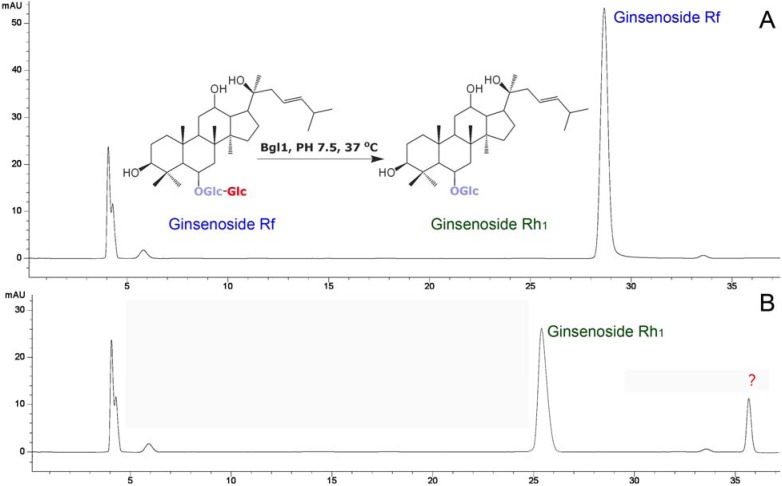
HPLC trace of enzyme assay. A: Trace of substrate ginsenoside Rf; B: Trace of enzyme assay of ginsenoside Rf reacted with BGL1.

Purified BGL1 protein was tested for biotransformation activities with ginsenoside Rf, DM_1_, PM_1_, SM_1_ [11] and compound K. The recombinant protein didn’t show any glucosidase activities towards compound K, ginsenosides DM_1_, PM_1_ and SM_1_ but did show the ability to transform ginsenoside Rf into new products, one of which has been identified as ginsenoside Rh_1_ by comparison of the retention time with the authentic compound and further confirmed by LC-MS analysis (Figure 3A and Figure 3B). 

## Conclusions

Ginsenoside Rh_1_ had been reported as a bioactive compound with various pharmacological effects [6,7,8,9], but the amount in the ginseng was relative minor. In the present study, a recombinant *Aspergillus niger* BGL1 protein showed the ability to transform ginsenoside Rf to Rh_1_, increasing the availability of this compound and hence its potential as a drug.

## Experimental

### General

HPLC runs were carried out on a Zorbax C_18_ column (150 x 25 mm, Phenomenex, Torrance, CA, USA) on an Agilent 1100 instrument and UV absorption data (λ_203_) were analyzed with Agilent Chemstation Ver 8.01. All solvents used in this study were HPLC grade, purchased from the Chinese Chemical Group, Beijing, P.R. China. *S. cerevisiae* MGY70 was used as host strain and the yeast shuttle vector pRS423 was used for the construct.

### Cloning and Expression of pRS-BGL1 in S. cerevisiae

Total RNA of overnight cultured *Aspergillus niger* was extracted using an RNAeasy mini kit, (Qiagen, USA). The full-length BGL1 cDNA was cloned using specific primers designed from the mRNA sequence deposited in GeneBank (Accession No. XM.001398779): sense primer 5'-GC *CTCGAG* ATGAGGTTCACTTCGATCGA-3' and antisense primer 5'- GC *GAATTC* TTAGTGAACAGTAGGCAGAG-3', with underlined nucleotides representing restriction sites included for *XhoI* and *EcoRI*. The PCR product was purified by a Mini-PCR purification kit (Invitrogen, USA), ligated into a pRS423 expression vector, sequenced and then introduced into *Saccharomyces cerevisiae*, selecting for growth on yeast nitrogen base (YNB) minimal medium (Difco) lacking histidine as appropriate.

### Purification of recombinant BGL1 protein

All steps were carried out at 4 ºC. His-tagged BGL1 protein was purified from the soluble fraction using a His-Bind purification kit (Novagen) following the manufacture’s protocol. Briefly, cells were freeze-thawed 3 times in binding buffer (500 mM NaCl, 20 mM Tris-HCl and 20 mM imidazole, PH 7.9). The suspension was incubated with lysozyme on ice for 30 min, and sonicated, the supernatant was collected by centrifugation at 14,000 g for 20 min and applied to pre-equilibrated His-Bind resin. Bound resin was washed three times with wash buffer (500 mM NaCl, 20 mM Tris-HCl and 60 mM imidazole, PH 7.9), then his–tagged protein was elute twice with three bed volumes of elution buffer (500 mM NaCl, 20 mM Tris-HCl and 1 M imidazole, PH 7.9), dialyzed three times against 1×PBS (140 mM NaCl, 2.7 mM KCl, 1.4 mM KH_2_PO_4_ and 8 mM Na_2_HPO_4_, PH 7.4) to remove immidazole and examined by SDS-PAGE on 13% denaturing gel.

### Enzyme Activity

To test the potential of BGL1 to catalyze the biotransformation of ginsenosides, *in vitro* enzyme assay conditions were altered to include incubation at 37 ºC for 12 h in 1 mL total volume containing 850 µL Tris-HCl buffer (100 mM, PH 7.0), 50µl purified BGl1 protein (0.9 µg µL^-1^ ), 100 µL ginsenoside (1 µg µL^-1^). Tested ginsenosides included ginsenosides Rf, DM_1_, PM_1_, SM_1_ and compound K. The reaction mixture with ginsenosides was centrifuged and subjected to HPLC for analysis (20 µL injection), at ambient temperature, a linear gradient of 5% to 65% Acetonitrile (containing 0.05% formic acid), (v/v) (flow rate of 1.0 mL/min), and monitored by PDA at A_203_. 
